# Contribution of Connexin Hemichannels to the Decreases in Cell Viability Induced by Linoleic Acid in the Human Lens Epithelial Cells (HLE-B3)

**DOI:** 10.3389/fphys.2019.01574

**Published:** 2020-01-20

**Authors:** Vania A. Figueroa, Oscar Jara, Carolina A. Oliva, Marcelo Ezquer, Fernando Ezquer, Mauricio A. Retamal, Agustín D. Martínez, Guillermo A. Altenberg, Aníbal A. Vargas

**Affiliations:** ^1^Instituto de Ciencias Biomédicas, Facultad de Ciencias de la Salud, Universidad Autónoma de Chile, Santiago, Chile; ^2^Instituto de Ciencias de la Salud, Universidad de O’Higgins, Rancagua, Chile; ^3^Department of Pediatrics, University of Chicago, Chicago, IL, United States; ^4^Centro de Envejecimiento y Regeneración (CARE-UC), Departamento Biología Celular y Molecular, Facultad de Ciencias Biológicas, Pontificia Universidad Católica de Chile, Santiago, Chile; ^5^Centro de Medicina Regenerativa, Facultad de Medicina, Clínica Alemana Universidad del Desarrollo, Santiago, Chile; ^6^Universidad del Desarrollo, Centro de Fisiología Celular e Integrativa, Facultad de Medicina Clínica Alemana, Santiago, Chile; ^7^Department of Cell Physiology and Molecular Biophysics, Texas Tech University Health Sciences Center, Lubbock, TX, United States; ^8^Center for Membrane Protein Research, Texas Tech University Health Sciences Center, Lubbock, TX, United States; ^9^Centro Interdisciplinario de Neurociencia de Valparaíso, Instituto de Neurociencia, Facultad de Ciencias, Universidad de Valparaíso, Valparaíso, Chile

**Keywords:** lens, connexin, polyunsaturated fatty acids, cell death, hemichannels

## Abstract

Connexin (Cx) proteins form gap junction channels (GJC) and hemichannels that a allow bidirectional flow of ions and metabolites between the cytoplasm and extracellular space, respectively. Under physiological conditions, hemichannels have a very low probability of opening, but in certain pathologies, hemichannels activity can increase and induce and/or accelerate cell death. Several mechanisms control hemichannels activity, including phosphorylation and oxidation (i.e., S-nitrosylation). Recently, the effect of polyunsaturated fatty acids (PUFAs) such as linoleic acid (LA), were found to modulate Cxs. It has been seen that LA increase cell death in bovine and human lens cells. The lens is a structure allocated in the eye that highly depends on Cx for the metabolic coupling between its cells, a condition necessary for its transparency. Therefore, we hypothesized that LA induces lens cells death by modulating hemichannel activity. In this work, we characterized the effect of LA on hemichannel activity and survival of HLE-B3 cells (a human lens epithelial cell line). We found that HLE-B3 cells expresses Cx43, Cx46, and Cx50 and can form functional hemichannels in their plasma membrane. The extracellular exposure to 10–50 μM of LA increases hemichannels activity (dye uptake) in a concentration-dependent manner, which was reduced by Cx-channel blockers, such as the Cx-mimetic peptide Gap27 and TATGap19, La^3+^, carbenoxolone (CBX) and the Akt kinase inhibitor. Additionally, LA increases intracellular calcium, which is attenuated in the presence of TATGap19, a specific Cx43-hemichannel inhibitor. Finally, the long exposure of HLE-B3 cells to LA 20 and 50 μM, reduced cell viability, which was prevented by CBX. Moreover, LA increased the proportion of apoptotic HLE-B3 cells, effect that was prevented by the Cx-mimetic peptide TAT-Gap19 but not by Akt inhibitor. Altogether, these findings strongly suggest a contribution of hemichannels opening in the cell death induced by LA in HLE-B3 cells. These cells can be an excellent tool to develop pharmacological studies *in vitro*.

## Introduction

Connexins are transmembrane proteins that form hexamers known as hemichannels. Docking of two hemichannels, each located in different neighboring cells, forms a GJC. Hemichannels and GJCs have different roles in cellular processes (Sáez et al., 2010). While hemichannels enable the flow of inorganic ions and molecules between intra and extracellular space (Sáez et al., 2010), GJCs mediate direct cytoplasmic communication, allowing a group of cells to elicit coordinated responses to a given stimulus (Warner, 1988; Herve and Derangeon, 2013). Due to that hemichannels are permeable to large molecules such as ATP and glutamate, it is well accepted that to prevent cell death they must have a low open probability (Contreras et al., 2002; Sáez et al., 2010). This notion is supported by the relationship between the hemichannels with high activity (leaky hemichannels) and the progression of several disorders, including cataracts, skin disorders, deafness, oculodentodigital dysplasia and the X-linked Charcot-Marie-Tooth disease (Abrams et al., 2002; Dobrowolski et al., 2008; Minogue et al., 2009; Retamal, 2014; Garcia et al., 2015; Retamal et al., 2015).

The lens is a transparent structure that focuses light on the retina. The lack of blood irrigation and organelles in the lens is essential for its transparency (Takemoto and Sorensen, 2008; Mathias et al., 2010). To survive the absence of blood flow, lens cells are coupled through GJCs formed by Cx43, Cx46, and Cx50 (Dobrowolski et al., 2008; Beyer and Berthoud, 2014), which allow diffusion of metabolites between cells located in the lens periphery and those located in the center (Mathias et al., 2010; Slavi et al., 2014). Although polyunsaturated fatty acids (PUFAs) exert beneficial effects to human health (Calo et al., 2013; Kar, 2013; Barrett et al., 2014), some PUFAs such as linoleic acid (LA) induce death of both bovine (Glaesser et al., 1996; Nguyen et al., 2000; Trimborn et al., 2000) and human lens epithelial cells *in vitro* (Iwig et al., 2004). Therefore, it has been proposed that a high PUFA dietary intake may affect the composition of lens lipid membrane, what would lead to develop nuclear opacity and cataracts. Indeed, patients with diabetes showed elevated levels of PUFAs in the aqueous humor (Trimborn et al., 2000; Iwig et al., 2004). Despite LA is a physiological constituent of the lens cell membranes, the exposure of human lens epithelial cell cultures to 10 μM LA induces alterations of intermediate filaments and bleb formation in the first 3 h; whereas higher doses like 50 μM LA inhibit protein-, RNA- and DNA-synthesis. However, the molecular mechanisms by which LA induces cell toxicity are not well understood (Iwig et al., 2004).

Since the massive opening of hemichannels can induce cell death (Retamal et al., 2015) and LA modulates the activity of hemichannels formed by Cx26, Cx43, and Cx46 (Retamal et al., 2011; Figueroa et al., 2013), we hypothesized that the effect of LA on the lens epithelial cells is the result of an abnormal activity of the hemichannels. Here, we explored whether HLE-B3 cells express functional hemichannels in the plasma membrane and whether these hemichannels are activated by LA. We found that HLE-B3 cells form functional hemichannels. Their activity rises in response to increasing concentrations of LA, as evaluated through dye uptake technique. Moreover, long exposure to high concentration of LA reduced HLE-B3 cell viability and increased the apoptotic cells, which was prevented by hemichannels blockers. Our results suggest that the massive opening of hemichannels is one of the underlying mechanisms of LA toxicity in lens epithelial cells.

## Materials and Methods

### Reagents

Lanthanum (La^3+^) chloride was obtained from Merck (Darmstadt, Germany), linoleic acid (LA), carbenoxolone (CBX), ethidium bromide (Etd^+^) were obtained from Sigma-Aldrich (St. Louis, MO, United States). The mimetic peptide Gap27 (SRPTEKTIFII) was synthesized by Anaspec (Fremont, CA, United States). The mimetic peptide TATGap19 (YGRKKRRQRRRKQIEIKKFK) was obtained from Tocris Bioscience (Bristol, United Kingdom.) Akt inhibitor VIII (AKTi) was obtained from Calbiochem (Merck, Darmstadt, Germany).

### Cell Culture

The HLE-B3 human lens epithelial cell line was obtained from ATCC (Rockville, MD, United States). Cells were cultured at 37°C and 5% CO_2_, in Dulbecco’s Modified Eagle Medium (DMEM), supplemented with 20% fetal bovine serum (FBS) (Life Technologies) plus 100 U/ml penicillin sulfate and 100 μg/ml streptomycin sulfate. The culture medium was replaced every 2 days, until cells reached 80% confluence. Attached cells were sub-culturing once reached 80% confluence, using trypsin-EDTA 0.25% (GIBCO, Invitrogen). In most experiments, the cells were seeded on round glass coverslips (#1, 12-mm radius, Marienfeld-Superior, Lauda-Königshofen, Germany). LA experiments were performed after 48 h of the last culture medium change, in order to get the maximum LA effect.

### Immunofluorescence

Human lens epithelial-B3 cells grown on glass coverslips were washed once with PBS (pH 7.4), fixed with 4% paraformaldehyde in PBS for 20 min, and permeabilized with 1% Triton X-100 for 10 min at room temperature. Non-specific antibody binding was blocked by incubation in PBS with 2% normal goat serum and 1% Triton X-100 for 1 h at room temperature. After fixation, permeabilization and blocking, cells were incubated overnight at 4°C with polyclonal antibodies (1:300, diluted in blocking solution) directed against human Cx43 (Invitrogen, Life Technologies, Carlsbad, CA, United States) or Cx46 (Santa Cruz Biotechnology) and monoclonal antibodies to αβ-crystallin (Santa Cruz Biotechnology) or Cx50 (Invitrogen, Life Technologies, Carlsbad, CA, United States). Cells were washed with PBS and incubated with goat anti-mouse IgG (H + L) secondary antibody; DyLight 488-conjugate and/or goat anti-mouse IgG (H + L) secondary antibody DyLight 594-conjugate (Pierce, Thermo Fisher Scientific Inc., Rockford, IL, United States). DAPI was used to detect nuclei in a fixed and permeabilized HLE-B3 cells. All images of immunostained HLE-B3 cells, were taken with a Nikon C1Plus confocal microscope using NIS-Elements acquisition software (Nikon, Tokyo, Japan).

### Western Blots

Human lens epithelial-B3 cell cultures were rinsed twice with PBS (pH 7.4) containing protease and phosphatase inhibitor ice-cold solution (# 11836153001, Roche) and harvested by scraping. Pelleted cells were resuspended in 60 μl of protease and phosphatase inhibitor fresh solution and lysed by sonication on ice using a Microson Ultrasonic Liquid Processor XL-2000 cell disrupter (Qsonica LLC, Newtown, CT, United States). Cell lysates (50 μg of protein) were resuspended in NuPAGE LDS 4X sample buffer (Novex, Life Technologies) containing 2.5% (v/v) β-mercaptoethanol (Sigma-Aldrich), then proteins were separated on a NuPAGE 10% Bis-Tris gel (Novex, Life Technologies) and electro-transferred to PVDF membranes. Non-specific proteins binding was blocked by incubation in buffer TBS containing 5% non-fat milk and 1% Tween-20 by 1 h. Afterward, blots were incubated overnight at 4°C with 1:1000 dilutions of polyclonal antibodies against human Cx43 (Life Technologies) or Cx46 (Santa Cruz Biotechnology), or a monoclonal antibody against human Cx50 (Life Technologies) or and αβ-crystallin (Santa Cruz Biotechnology). Then, the membranes were washed five times (20-minute each) with TBS containing 1% Tween-20. After washing, membranes were incubated with a 1/5000 dilution of a horseradish peroxidase-conjugated goat anti-rabbit antibody (Pierce, Thermo Fisher Scientific) or a horseradish peroxidase-conjugated goat anti-mouse antibody (Novex, Life Technologies). Proteins were visualized by chemiluminescence using the SuperSignal West Femto reagent (Pierce, Thermo Fisher Scientific) and detected on a C-DiGit Blot Scanner (LI-COR, Lincoln, NE, United States). After analysis, immunoblots were washed briefly and were incubated with a mouse monoclonal beta-Tubulin monoclonal antibody (1:5000; Pierce, Thermo Fisher Scientific Inc., Rockford, IL, United States) for 1 h at room temperature (loading control), followed by horseradish peroxidase conjugated with goat anti-mouse antibody. Beta-tubulin was detected as described above.

### Dye Uptake Assay

Hemichannel activity was evaluated through the uptake of Etd^+^ (charge = + 1, MW = 394). For each experiment, HLE-B3 cells were seeded at ∼70% confluence onto glass coverslips and used 48 h later. For all experiments, a single coverslip was placed in a 35 mm plate and bathed in a recording solution (in mM: NaCl, 140; KCl 4; CaCl_2,_ 2; MgCl_2_, 1; glucose 5; HEPES 10; pH 7.4) which contained 5 μM Etd^+^. Etd^+^ fluorescence intensity was measured using an inverted microscope (Eclipse Ti- U, Nikon). Images were captured with a high-sensitivity cooled monochrome camera (CFW-1310M CCD DS-Qi1, Nikon) at 30-s intervals. To increase hemichannels opening, the recording solution was replaced by a solution without Ca^2+^ and Mg^2+^ (divalent cation-free solution, DCFS). For fluorescence intensity analysis, regions of interest (ROI) were defined by the cell nuclei. The dye uptake rate was calculated from the fluorescence intensity from captured images using the NIS-elements advanced research software (version 4.0, Nikon). The fluorescence intensity of at least 30 cells per experiment was averaged and plotted against time; the slope (which represent the rate of Etd^+^ influx) was calculated with GraphPad Prism (version 6.03) software (GraphPad Software, San Diego, CA, United States). We have previously shown that under control conditions the increase in Etd^+^ fluorescence is nearly linear with time for more than 20 min, therefore it is used as an indication of unidirectional cellular influx (Retamal et al., 2011; Figueroa et al., 2013).

### Extracellular ATP Measurement

The release of ATP from HLE-B3 cells was evaluated as previously described (Figueroa et al., 2014). Briefly, cells were seeded into 60-mm diameter dishes at 70% confluence and 48 h later they were washed once with DCFS and then 500 μl of the same fresh solution were added. ATP released after 5-minute incubation was determined by luminescence using the ATP determination kit (Life Technologies) following the manufacturer’s instructions. ATP-associated bioluminescence was measured with a spectrofluorometer (Jasco FP-63000, Tokyo, Japan).

### Intracellular Calcium Signal Measurement

The ratiometric calcium indicator Fura-2 AM (membrane-permeant derivative of the ratiometric calcium indicator Fura-2) was used to visualize changes in intracellular free-calcium signal (hereinafter termed the Ca^2+^ signal), as previously described (Vargas et al., 2017). In brief, cells seeded on glass coverslips were loaded for 30 min at 37°C with 5 μM Fura-2AM (Invitrogen, MA, United States) in the same saline solution used for the dye uptake assay and were then washed with the same solution without Fura-2AM. For Ca^2+^ signal measurements, fluorescence intensity was captured every 3 s. Images and the fluorescence intensity ratio quantification (Ca^2+^ signal = F340/F380) were performed in a Nikon Eclipse Ti inverted microscope using NIS-Elements software (Nikon, Tokyo, Japan).

### Cell Viability

Cell viability was measured using the Resazurin cell viability assay (Sigma-Aldrich). Resazurin is a blue non-fluorescent dye cell-permeable, which is reduced to resorufin upon entering the cells, yielding a pink-fluorescent product. Viable cells an active metabolism continuously convert resazurin to resorufin, and the resulting fluorescence intensity provides a quantitative measure of cellular viability. For this assay, HLE-B3 cells were seeded into 24-well plates (5000 cells *per* well), in DMEM supplemented with 20% FBS and cultured for 48 h at 37°C in a humidified atmosphere containing 5% CO_2_. Afterward, HLE-B3 cells were treated for 2 h with different concentrations of LA, with or without 100 μM CBX, a non-selective both GJC and hemichannel blocker (D’Hondt et al., 2009). For the estimation of viable cells, LA and CBX were removed by washing cells twice with PBS, and then 30 μl of reagent (0.15 mg/ml) was added to each well containing DMEM. After a 4-hour incubation at 37°C, fluorescence was recorded at 590 nm with excitation at 530 nm using a Multi-Mode Microplate Reader (Synergy HT). As a positive control for cell death, HLE-B3 cells were incubated with hydrogen peroxide (H_2_O_2_, 1 mM) for 4 h at 37°C. Results were analyzed plotting resorufin fluorescence intensity vs. compound concentration.

### Apoptosis Assay

Apoptotic or necrotic cell death was determined by using Pacific Blue-Annexin V/PI Apoptosis Detection Kit Cell (Pacific Blue^TM^ BioLegend, San Diego, CA, United States). Briefly, HLE-B3 cells were grown on cover slips to confluence in 6 well tissue culture plates and treated with 20 and 50 μM of LA with or without TATGap19 or AKT inhibitor for 2 h. After treatment cells were washed twice with PBS and Annexin V and Propidium iodide (PI) solution were then added to stain the cells before analysis according to kit’s instructions. After staining, at least five randomly picked microscopic fields were examined under a fluorescence microscope for each condition. Five images (in each culture) were taken using a Nikon Eclipse Ti inverted microscope equipped with a × 10 objective (Nikon, Tokyo, Japan) and high-sensitivity cooled monochrome camera (CFW-1310M CCD DS-Qi1, Nikon). The number of cells being Annexin V positive and propidium iodide negative (apoptotic cells), the number of cells being both Annexin V and propidium iodide positive (necrotic cells) and the total cell number, were counted in each image using ImageJ (Bethesda, MD, United States) and expressed relative to the number of nuclei present and stated as the Annexin V + cells (%).

### Statistics

Statistical analysis was performed using GraphPad Prism 5 for Windows (GraphPad Software). Data sets (means ± SEM) were compared using one-way analysis of variance (ANOVA) followed by a Tukey’s *post hoc* test.

## Results

### Expression of Cx43, Cx46, and Cx50 in HLE-B3 Cells

**FIGURE 1:**
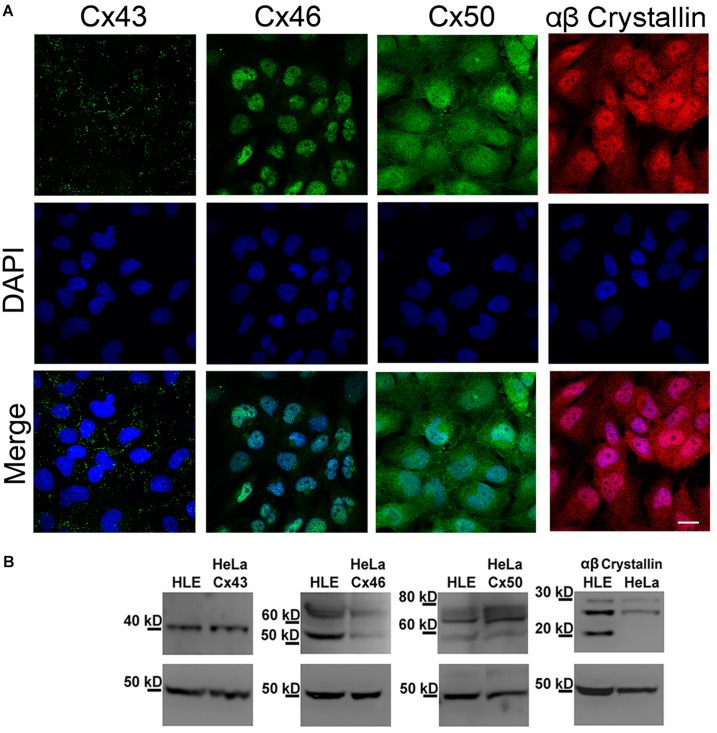
Expression of Cx43, Cx46, Cx50 and αβ-crystallin in HLE-B3 cells. **(A)** Indirect immunofluorescence. Upper panels: representative indirect immunofluorescence confocal micrographs showing the subcellular localization of Cx43, Cx46, Cx50 (green) and αβ-Crystallin (red). Middle panels: DAPI (blue), used to detect nuclei of the cells in the upper panel. Bottom panels: merge of DAPI and immunofluorescence staining. Scale bar 25 μm. **(B)** Immunoblots from total homogenates of the HLE-B3 cell line (HLE) and HeLa cells (HeLa) transfected with human Cx43, Cx46 or Cx50, used as positive controls. β-tubulin was used as loading control.

We performed indirect immunofluorescence analyses to determine whether HLE-B3 cells express the Cx isoforms normally expressed in lens epithelial cells (Cx43, Cx46, and Cx50) (Mathias et al., 2010). Cx43 showed the typical punctate staining pattern indicative of gap junction plaques located in the plasma membrane of adjacent cells (Figure 1A, Cx43 green dots), which was also confirmed by TIRF microscopy (Supplementary Material). In the case of Cx46 and Cx50, the immunostaining shows a sparse and tenuous staining, observed mainly in the cytoplasm and in the nuclear region; no staining in regions of close apposition of the plasma membranes was observed (Figure 1A, Cx46 and Cx50). HLE-B3 cells also express αβ-crystallin, a heat shock protein expressed preferentially in the lens (Andley et al., 1994). The αβ-crystallin was uniformly distributed in the cytoplasm in a diffuse pattern and in most of the cells, a nuclear staining was also evident (Figure 1A, αβ-crystallin). We also performed western blot analyses to confirm the presence of Cxs and αβ-crystallin in HLE-B3 cells. Total cellular extracts from HeLa cells transfected with human Cx43, Cx46, or Cx50 were used as positive controls. We observed a single band near 40 kDa, corresponding to Cx43, two bands (50 kDa and 60–70 kDa) corresponding to Cx46, and three bands (between 60 and 80 kDa) corresponding to Cx50 (Figure 1B). It is likely that the smaller and larger Cx46 and Cx50 bands correspond to nonphosphorylated and phosphorylated forms, respectively (White et al., 1992; Koval et al., 1997; Banerjee et al., 2011). The three αβ-crystallin bands between 20 and 30 kDa corresponds to a full-length form and two truncated forms in the C-terminal, as has been previously reported (Brady et al., 2001).

### HLE-B3 Cells Express Functional Hemichannels Which Mediated Ethidium-Uptake

**FIGURE 2:**
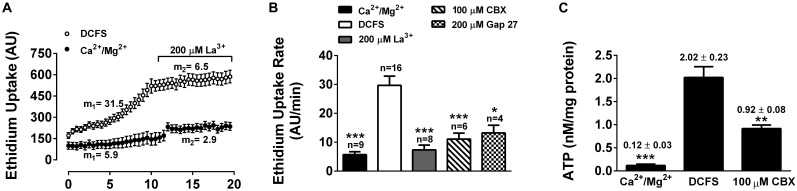
Functional connexin-hemichannels in HLE-B3 cells. Ethidium (Etd^+^, 5 mM) uptake in cells bathed with solution containing divalent cations (Ca^2+^/Mg^2+^) or with divalent cation-free solution (DCFS). **(A)** Etd^+^ uptake time lapse (fluorescence intensity in arbitrary units, AU) under control (black circles) and DCFS (white circles) conditions. During the last 10 min of recording the cells were exposed to 200 μM La^3+^. Measurements were taken every 30 s for 20 min. Each value corresponds to the mean ± SEM of at least 30 cells. The average slopes in the absence (m_1_) and presence of La^3+^ (m_2_) are shown in AU/min. **(B)** Etd^+^ uptake rates of HLE-B3 cells under either control condition (Ca^2+^/Mg^2+^), DCFS, after acute exposure of cells to La^3+^ (200 μM) or pre-incubated with CBX: carbenoxolone (100 μM) or Gap27: Cx-mimetic peptide (200 μM) in DCFS. Each bar represent mean ± SEM, with the number of independent experiments indicated on top of the bars. ^∗∗∗^*P* < 0.001 and ^∗^*P* < 0.05 for DCFS vs. all conditions respectively. **(C)** ATP-released from HLE-B3 cells in response to DCFS. ATP levels was determined with an ATP bioluminescence assay under control condition (Ca^2+^/Mg^2+^) and after exposure to DCFS (5 min) in the absence or presence of 100 μM CBX. The data are means ± SEM of three independent experiments. ^∗∗^*P* < 0.01; ^∗∗∗^*P* < 0.001.

To determine whether Cxs expressed in HLE-B3 cells form functional hemichannels, we measured influx of Etd^+^ and ATP efflux, under conditions that are known to increase hemichannels activity. Etd^+^ is a positively charged dye that upon binding to DNA increases its quantum yield fluorescence drastically. Etd^+^ permeability across cell membranes is very low, but it can permeate through open hemichannels. Indeed, there is a good correlation between Etd^+^ uptake and hemichannels activity (Sáez et al., 2003). In the presence of physiological concentrations of Ca^2+^ and Mg^2+^, HLE-B3 cells showed a slow rate of Etd^+^ uptake (Figure 2A, filled dots) that increases when cells were exposed to a divalent cation-free solution DCFS (Figure 2A, empty dots), a condition that increase hemichannels open probability (Verselis and Srinivas, 2008). The extracellular addition of 200 μM lanthanum (La^3+^), a non-specific hemichannel blocker, decreased Etd^+^ uptake rate in the HLE-B3 cells in both conditions, control and in DCFS (Figures 2A,B). Pre-incubation with CBX or the mimetic peptide Gap27 for 20 min, reduced the Etd^+^ uptake induced by DCFS (Figure 2B). The similar effect of the three hemichannel blockers suggests that most of the Etd^+^ uptake in DCFS occurs through Cx channels. However, La^3+^ and CBX are non-specific blockers and Gap 27 does not discriminate between hemichannels and GJC (D’Hondt et al., 2009).

It is well known that ATP diffuses through open hemichannels, which constitutes an important paracrine signaling pathway (Cotrina et al., 1998; De Vuyst et al., 2007). Therefore, we evaluated the release of ATP from HLE-B3 cells. In the presence of divalent cations, the extracellular concentration of ATP in HLE-B3 cell cultures was very low or almost undetectable (Figure 2C). After 5 min of exposure to DCFS, the extracellular ATP increased by about 17-fold, which was prevented significantly by the presence of CBX 100 μM. This result is consistent with hemichannels mediated-ATP efflux. Altogether, these data strongly suggest that HLE-B3 cells present functional hemichannels at their plasma membrane, which can mediate the transport of small hydrophilic compounds such as Etd^+^ and ATP.

### Linoleic Acid Induces Hemichannels Opening in HLE-B3 Cells

**FIGURE 3:**
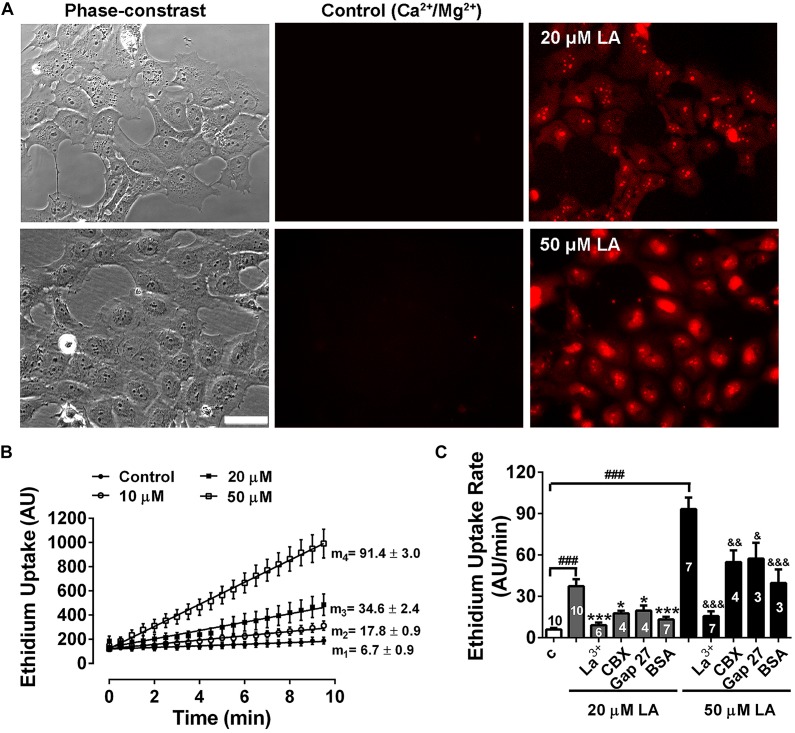
Linoleic acid induces hemichannels opening in HLE-B3 cells. Ethidium (Etd^+^, 5 mM) uptake under control conditions and during the exposition to linoleic acid (LA). **(A)** Representative images show a time-dependent increase in Etd^+^ fluorescence, under control condition (Ca^2+^/Mg^2+^) and after exposure to 20 and 50 μM LA (10 min). Scale bar 50 μm. **(B)** Representative real-time Ethidium (Etd^+^, 5 μM) uptake in HLE-B3 cells bathed with solution containing divalent cations (Control) and after addition of different concentrations of LA (10, 20, and 50 μM). The values are presented as mean ± SEM of at least 30 cells; m_1_, m_2_, m_3_, m_4_ = average slopes. **(C)** The effect of connexin channel blockers on Etd^+^ uptake rates LA-induced in HLE-B3. La^3+^ (200 μM), CBX (Carbenoxolone, 100 μM), Gap 27 (synthetic connexin 43-mimetic peptide, 200 μM), BSA (bovine serum albumin, 1 mM). The data represent mean ± SEM, with the number of independent experiments indicated in each bar. ^∗∗∗^*P* < 0.001 and ^∗^*P* < 0.05 for 20 μM LA vs. all blockers in presence of 20 μM LA; ^&^*P* < 0.05, ^&&^*P* < 0.01 and ^&&&^*P* < 0.001 for 50 μM LA vs. all blockers, respectively in presence of 50 μM LA; ^###^*P* < 0.001 for control condition vs. 20 and 50 μM LA.

**FIGURE 4:**
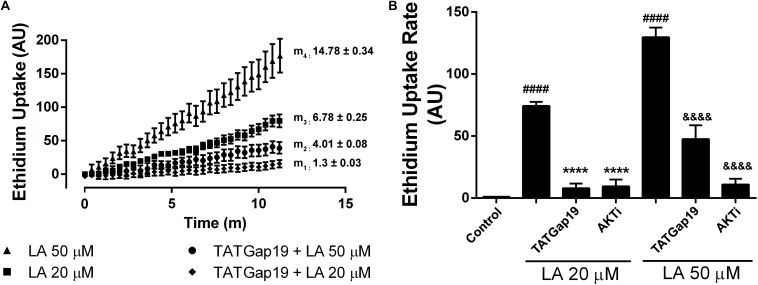
Inhibition of either Cx43 hemichannels or Akt kinase reduced the Etd^+^ uptake increase induced by linoleic acid in HLE-B3 cells. **(A)** Representative real time Etd^+^ uptake (fluorescence, AU) in HLE-B3 cells induced by 20 μM and 50 μM LA with or without TATGaP 19 (100 μM). The values represent means ± SEM of at least 30 cells; m_1_, m_2_, m_3_, m_4_ = average slopes. **(B)** Etd^+^ uptake rates (AU/min) LA-induced in HLE-B3 cells in the absence and presence of TATGap19 (100 μM) or AKT VIII inhibitor (AKTi, 10 μM). The data represent mean ± SEM, of three independent experiments to each condition. ^####^*P* < 0.0001 control vs. 20 and 50 μM LA; ^∗∗∗∗^*P* < 0.0001 20 μM LA vs. TATGap19 or AKTi plus 20 μM LA; ^&&&&^*P* < 0.0001 50 μM LA vs. TATGap19 or AKTi plus 50 μM LA.

Previously, we have shown that LA induces opening of hemichannels formed by both Cx46 in *Xenopus laevis* oocytes, and by Cx43 in HeLa cells (Retamal et al., 2011; Figueroa et al., 2014). To test whether LA increases hemichannels activity in HLE-B3 cells, we determined the effects of acute exposure to this fatty acid using the Etd^+^ uptake assay. Under control conditions (normal Ca^2+^/Mg^2+^), HLE-B3 cells showed a low rate of Etd^+^ uptake. Exposure to increasing concentrations of LA (10, 20, or 50 μM) produced an increase in Etd^+^ uptake in a concentration-dependent manner (Figures 3A,B). The addition of 200 μM La^3+^ or pre-incubation for 20 min with 100 μM CBX or 200 μM Gap27 reduces Etd^+^ uptake induced by LA (Figure 3C). This is consistent with the idea that the increased Etd^+^ uptake induced by LA occurs mainly through hemichannels. Moreover, BSA 1 mM reduced the Etd^+^ uptake induced by LA around ∼60% (Figure 3C). Since BSA acts as the main fatty acid binding protein in extracellular fluids, this data is consistent with the evidence that is LA what causes the opening of hemichannels in HLE-B3 cells. To confirm this hypothesis, we used the Cx43 mimetic peptide TATGap19, a specific hemichannel blocker, which has no effect on GJC (Ponsaerts et al., 2010; Wang et al., 2013; Abudara et al., 2014). TATGap19 reduced significantly the Etd^+^ rate uptake induced by 20 or 50 μM LA (Figures 4A,B), strongly suggesting that is mediated by hemichannels.

In order to elucidate the signaling involved in this response, we evaluated the well-known effect of Akt on connexins. As has been previously shown, Akt-dependent phosphorylation of connexin 43 increases hemichannels activity (Salas et al., 2015). Moreover, the cell-permeable AKTi, which inhibits Akt1/Akt2 pathway, reduces the Etd^+^ uptake rate induced by LA in HeLa-Cx26 cells (Figueroa et al., 2013). Therefore, we tested the effect of this inhibitor on HLE-B3 cells. Pre-incubation of these cells with 10 μM AKTi by 20 min, drastically reduces the Etd^+^ uptake rate induced by LA (Figure 4B). This is consistent with the expression of functional Cx43-hemichannels distributed on the plasma membrane of HLE-B3 cells.

### Linoleic Acid Increases Intracellular Ca^2+^ Levels in HLE-B3 Cells, Through the Opening of Cx43-Hemichannels

**FIGURE 5:**
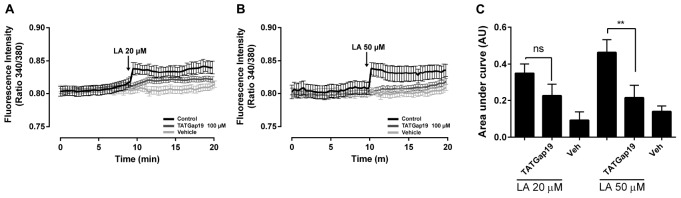
The LA-induced rise in intracellular Ca^2+^ signal in HLE-B3 cells is sensitive to HC blockade. HLE-B3 cells were loaded with 5 μM Fura-2AM in serum-free medium for 30 min. **(A,B)** Representative average tracings (including 30 cells) showing the Concentration-dependent time course of Linoleic acid (LA)-evoked Ca^2+^ signals, in the absence or presence of TAT Gap19 (100 μM) and LA vehicle. The data represent mean ± SD. **(C)** Compiled data show the amplitude of Ca^2+^ signal changes evoked by extracellular LA in presence or absence of TAT Gap19 and vehicle. Each bar represents the area under the curve [AUC in Arbitrary Units (AU)], which was measured with a base line since the first Ca^2+^ signal in each curve, in at least 30 cells, in three independent experiments as shown in **A** and **B**. The values represent the mean ± SD of the three independent experiments ^∗∗^*P* < 0.01 (Veh, vehicle). All experiments were conducted in the presence of physiological concentrations of divalent cations.

It has been described that Cx43 hemichannels are permeable to Ca^2+^ (Schalper et al., 2010). On the other hand, LA increases the free intracellular Ca^2+^ concentration ([Ca^2+^]_*i*_) in HeLa Cx26 and this increase requires Ca^2+^ inflow via hemichannels (Figueroa et al., 2013). Therefore, we tested whether LA affects intracellular Ca^2+^ signal in HLE-B3 cells. Extracellular LA application (20 or 50 μM) induced a fast and transient rise of intracellular Ca^2+^ signal, followed by a progressive and sustained increase (Figures 5A–C). Preincubation with TATGap19 for 20 min, reduced both transient and sustained Ca^2+^ signal rise, suggesting that this effect require the opening of Cx43-hemichannels (Figures 5A–C).

### Linoleic Acid Reduces HLE-B3 Cell Viability Through Modulation of Connexin Channels Activity

**FIGURE 6:**
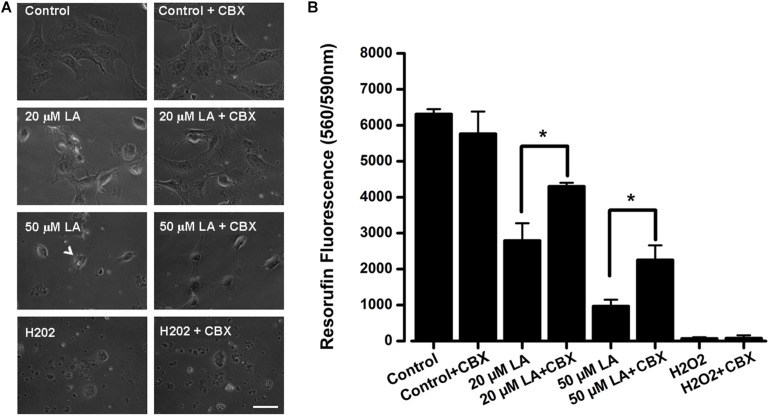
Dose-dependent decrease in HLE-B3 cell viability induced by Linoleic acid. **(A)** Representative images of HLE-B3 cells incubated by 2 h in control condition (Control), with 20 or 50 μM LA, and Hydrogen peroxide (H_2_O_2_), in the absence or presence of 100 μM CBX. **(B)** HLE-B3 cell viability in control conditions (Control), with 20 or 50 μM LA, and Hydrogen peroxide (H_2_O_2_), in the absence or presence of 100 μM CBX (CBX). Cell viability was measured by the Resazurin/resorufin assay and expressed as resorufin fluorescence at 560 nm/590 nm (*n* = 3). Hydrogen peroxide (H_2_O_2_, 1 mM) was used as cell toxicity positive control. Scale bar 50 μm. ^∗^*P* < 0.05, 20 or 50 μM LA vs. 100 μM CBX plus LA, respectively.

Previous work has shown that LA induces cell death of human and bovine lens epithelial cells (Glaesser et al., 1996; Nguyen et al., 2000; Trimborn et al., 2000; Iwig et al., 2004), but the mechanism behind this phenomenon is poorly understood. Here, we tested whether LA affects the viability of HLE-B3 cells and if this effect depends on connexin channels activity. The extracellular addition of LA reduced cell viability in a concentration-dependent manner, as was determined by Resazurin/resorufin assay (Figure 6A). When HLE-B3 cells were left in a culture medium (DMEM) without FBS for 2 h, no changes in cell morphology were observed (Figure 6A, control). Similarly, when HLE-B3 cells were exposed to 100 μM CBX in DMEM without FBS for 2 h, no overall changes were noticed. However, when 20 μM of LA was added to the culture media, morphological changes in the HLE-B3 cells were observed. Among them, an increased number of spherical cells and the reduction in cell adhesion capacity resulting in cell death. All these effects were prevented by preincubation with 100 μM CBX (Figure 6A, second line of the panel). We performed the same experiment using 50 μM LA for 2 h. We found more cells with spherical form, cell shrinking and also “arborization” of some cells, together with a massive loss in cell adhesion (Figure 6A, arrow, third line of the panel). This is consistent with previous reports showing that LA induces cell damage and morphological changes in bovine and human lens epithelial cells, characterized by shrinkage, rounding and reduced adhesion (Glaesser et al., 1996; Nguyen et al., 2000; Trimborn et al., 2000; Iwig et al., 2004). Indeed, in our experiments the exposure to LA reduced cell viability around 51% with 20 μM and 80.5% with 50 μM (Figure 6B). These effects were partially inhibited by the pre- incubation with 100 μM CBX (Figure 6B). Additionally, we observed that CBX did not prevent cell death induced by 1 mM H_2_O_2_ (Figure 6A, fourth line in the panel and Figure 6B), indicating that CBX protects specifically against cell death induced by the LA through connexin-channels.

**FIGURE 7:**
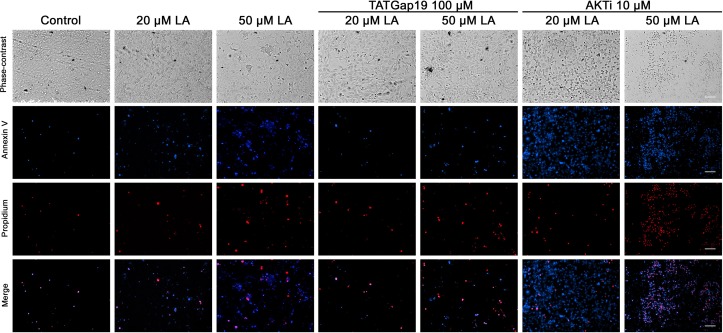
Linoleic acid induces HLE-B3 cell apoptosis. Representative images Annexin V-Pacific Blue and Propidium iodide fluorescence double staining, showing HLE-B3 cell apoptosis after 20 and 50 μM Linoleic acid treatment by 2 h, with or without 100 μM TATGap19 or 10 μM AKTi. Upper panel: phase contrast. Second panel: Fluorescence microscopy images of Annexin V-Pacific Blue; Third panel: Fluorescence microscopy images of Propidium iodide. Scale bar: 100 μm.

**FIGURE 8:**
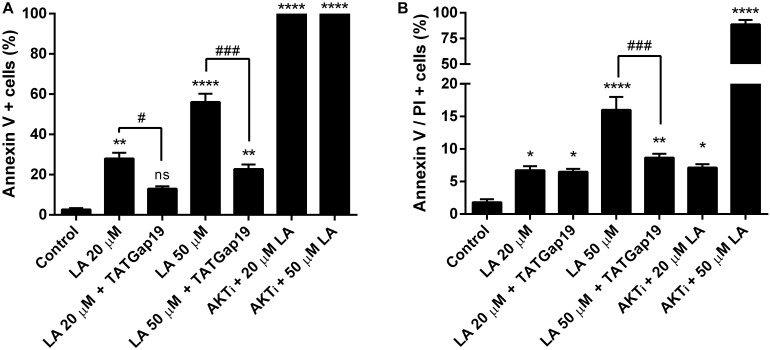
Inhibition of Cx43-hemichannels, decrease the Linoleic acid-induced apoptosis in HLE-B3 cells. **(A)** Percentage of Annexin-V positive cells detected after 2 h Linoleic acid treatment combined or not, with of TATGap19 or AKTi. Annexin-V positive, but PI negative cells were counted under a fluorescence microscopy. The data are shown as the mean ± SEM obtained from five randomly picked microscopic fields for each condition. ^∗∗^*P* < 0.01, ^∗∗∗∗^*P* < 0.0001 and ns (no significant) between control group v/s all groups. #*P* < 0.05 and ###*P* < 0.001 significant differences between 20 and 20 μM LA + 100 μM TATGap19 and 50 and 50 μM LA + μM 100 TATGap19, respectively. **(B)** Percentage of double stained cells by Annexin V and propidium iodide after 2 h Linoleic acid treatment combined or not, with of TATGap19 or AKTi. The data are shown as the mean ± SEM obtained from five randomly picked microscopic fields for each condition. ^∗^*P* < 0.05, ^∗∗^*P* < 0.01, and ^∗∗∗∗^*P* < 0.0001 significant differences between control condition compared with all group. ^###^*P* < 0.001 significant differences between 50 and 50 μM LA + 100 μM TATGap19 group. Data is show as a percentage of all cells in the field.

Since a growing body of evidence shows that connexin-channels modulate apoptosis, we tested the apoptotic effect of the opening of hemichannels in response to LA. To do this, we performed an Annexin V assay for the determination of phosphatidylserine residues exposure, one of the earlier steps involved in the apoptotic process (Shi et al., 2018), both in the presence or absence of 100 μM TATGap19, the specific Cx43 hemichannel inhibitor. When HLE-B3 cultures were treated with LA 20 or 50 μM by 2 h, the Annexin V positive cells (early apoptosis) increased by 28 and 56%, respectively compared to the control (relative to the total cells in the field) (Figures 7, 8A). Meanwhile, in combination with TATGap19 100 μM by 2 h, Annexin V labeling was consistently low in HLE-B3 cells exposed 20 or 50 μM of LA (Figures 7, 8A). This suggests that an apoptotic initiation process is caused by LA through hemichannels.

On the other hand, since PI3K/Akt pathway regulates cell viability and apoptosis in many cell types, we evaluated how the specific pharmacological inhibition of Akt affects the HLE-B3 cell viability. The treatment with 20 or 50 μM of LA combined with AKTi 10 μM evidenced a massive cell death (Figures 7, 8A). The proportion of late apoptotic and necrotic HLE-B3 cells, stained by both PI and Annexin V, significantly increased after treatment with 20 μM LA. However, the co-incubation with TATGap19 or AKTi do not modify this proportion (Figures 7, 8B). Instead, the proportion of late apoptotic and necrotic HLE-B3 cells after the treatment with LA 50 μM, is three-times the observed with 20 μM LA, and the co-incubation with TATGap19 100 μM reduces significantly this effect (Figures 7, 8B). These results are consistent with the role of PI3K/Akt pathway, where it’s blockage affects lens epithelial cells survival (Xiao et al., 2010; Liegl et al., 2014).

## Discussion

Our results show that the exposure of HLE-B3 cells to high levels of extracellular LA, resulted in a marked decrease in cell viability, indication of cell death induction. Our data indicate that this effect appears to be related to the capacity of LA to enhance hemichannels opening, especially those formed by Cx43 at the cell membrane. Using immunofluorescence microscopy, we observed that Cx43 was present in regions of close apposition of the plasma membranes of adjacent HLE-B3 cells, with punctate staining pattern, which is characteristic of gap-junction plaques (Falk, 2000). Cx46 and Cx50 immunoreactivity was mostly located in the perinuclear zone and cytoplasmic compartments; although the presence of hemichannels at the plasma membrane and the formation of small gap junction plaques cannot be ruled out (Falk, 2000). These results are in agreement with previous studies showing Cx43 gap junction plaques in HLE-B3 cells (Yao et al., 2008) and co-expression of Cx43, Cx46 and Cx50 in the human lens epithelial cells (Banerjee et al., 2011). In contrast, other studies have revealed that both Cx43 and Cx50 form gap junction plaques in mouse lens epithelial cells, whereas Cx46 is absent (White et al., 2007). Independently of what isoforms are present and what is their location, the evidence of functional hemichannels in human lens epithelial cells *in vitro* has not been reported yet. Here, we demonstrated that HLE-B3 cells are permeable to Etd^+^, whose rate of uptake was enhanced when cells were exposed to conditions known to increase hemichannels open probability (DCFS). Moreover, acute exposure to hemichannel blocker La^3+^ or the preincubation with CBX or Gap27 significantly decreased the DCFS-induced Etd^+^ uptake rate. Although La^3+^ and CBX are non-specific hemichannels and GJC blockers, their effects were similar to those of Gap27, a specific hemichannels and GJC blocker (D’Hondt et al., 2009). Indeed, to our knowledge connexin channels are the only channels inhibited by extracellular divalent cations, La^3+^ and Gap27. Furthermore, we observed that extracellular ATP concentration increase in HLE-B3 cells in response to DCFS, an effect that was significantly reduced by CBX, which is consistent with ATP efflux through hemichannels (Kang et al., 2008; Maes et al., 2017).

On the other hand, we found that exposure to LA increases HLE-B3 cells Etd^+^ uptake rate, which also was prevented by La^3+^, CBX, and Gap 27. Furthermore, using TAT-Gap19 peptide, a specific Cx43-hemichannel inhibitor, which has no significant affinity for gap junctions or Pannexin1 channels, we demonstrated that the effect of LA was mediated by hemichannels composed of Cx43. Therefore, our data strongly suggests the presence of functional Cx43 hemichannels in these cells (Schalper et al., 2009).

LA has been shown to induce deleterious effects in a variety of cell types (Cury-Boaventura et al., 2004; Choi, 2014; Brown et al., 2018). In our work, we demonstrated that LA induced both hemichannels opening and reduction of cell viability in HLE-B3 cells, nevertheless the molecular mechanism is unknown. According to the results, HLE-B3 cells treated with LA resulted in high proportion of apoptotic cells. Moreover, Cx43-hemichannel blocker TATGap19 inhibited apoptosis induced by LA by more than 50%, suggesting that Cx43-hemichannels are involved. Although TATGap19 significantly reduces cell death induced by LA, does not completely prevent it, and GJC participation cannot be ruled out. Besides that, the reduction in HLE-B3 cell viability was partially prevented with CBX, which block both GJC and hemichannels. Indeed, we found that HLE-B3 cells are dye-coupled, because the transference of Lucifer yellow (LY) and Neurobiotin (NB) was inhibited when cells were treated with 18β-glycyrrhetinic acid, a GJC-blocker (see Supplementary Figure S2), indicating that HLE-B3 cells are also coupled through GJC. Though, in our experiments we cannot differentiate between the role of hemichannels and GJC in the apoptosis induced by LA in HLE-B3 cells and more studies are needed to address this issue.

To this point, we cannot rule out the participation of Cx46 and Cx50 as LA-direct or indirect signaling targets, however, Cx43 appears to be most likely involved. The Gap27 is more selective to Cx43 than other isoforms (D’Hondt et al., 2009) and TATGap19 inhibits specifically hemichannels composed of Cx43 (Ponsaerts et al., 2010; Wang et al., 2013; Abudara et al., 2014). Moreover, immunofluorescence and TIRF analyses shown that is Cx43, but no Cx46 and Cx50, which is clearly present in the plasma membrane of HLE-B3 cells (Supplementary Figure S1). Therefore, the reduced viability of HLE-B3 cells induced by LA, is the result of hemichannels opening, which is consistent with previous observations showing that massive hemichannels opening can damage cells or induce cell death (Retamal et al., 2015; Salas et al., 2015). Previous studies, in HeLa and C6 glioma cells models, suggest that the expression of the Cx, and in particular Cx43, increases the proportion of late apoptotic and necrotic HeLa cells. This effect that depends on the ability of Cxs to form functional GJC and hemichannels, causes that proapoptotic signal transfers between cytoplasms of adjacent cells, or from the intracellular to the extracellular space, or vice versa (Hur et al., 2003; Kalvelyte et al., 2003; Decrock et al., 2009). Moreover, previous studies show Akt-dependent increase in Cx43 hemichannels activity in HeLa cells and in cortical astrocytes under metabolic inhibition (Salas et al., 2015). Besides, LA induces connexin-hemichannels activity in both HeLa-Cx43 and MKN28 cells, via a GPR40- and Akt-dependent mechanism (Puebla et al., 2016). We have also previously shown that specific PI3K/Akt inhibitors reduce the hemichannel activity induced by LA in HeLa-Cx26 cells (Figueroa et al., 2013). In this study, we observed that hemichannels activity induced by a brief exposition (10 min) of HLE-B3 cells to LA, was reduced by the specific AKTi, however, after a long time exposure to LA (2 h), AKTi inhibitor did not prevent the increase of apoptotic cells, indeed, results showing a cell death-enhancing effect by the Akt inhibition, suggesting that Akt activity is important for HLE-B3 cell survival. Previous studies have been shown that the Akt signaling pathway plays a pivotal role in proliferation, migration and survival of human lens epithelial cell lines, including HLE-B3 cells, were the inhibition of active Akt form, by specific dephosphorylation, reduce the cell viability of lens epithelial cells and retinal pigment epithelial cells under pro-apoptotic stimulus (Xiao et al., 2010; Liegl et al., 2014), however, we still need to determine whether the increase of hemichannels activity and cell death induced by LA is due to the same signaling pathway.

How do the hemichannels induce cell death? It has been suggested that a massive hemichannel opening can result in large efflux of amino acids (Stridh et al., 2008) and ATP (Stout et al., 2002), as well as intracellular Ca^2+^ overload partially mediated by Ca^2+^ influx through hemichannels (Sánchez et al., 2010; Schalper et al., 2010). We previously reported that in HeLa cells that express Cx26, LA induces an increase in the free intracellular Ca^2+^ concentration, mediated by Ca^2+^ influx through Cx26-hemichannels (Figueroa et al., 2013). Here, we have shown a similar increase in free intracellular Ca^2+^ concentration in HLE-B3 cells in response to LA, which was significantly reduced by pre incubation with TAT-Gap19. This suggests that Cx43 hemichannels are involved. As has been previously reported, a noxious stimuli like metabolic inhibition, activation of Akt pathway, increase in intracellular Ca^2+^ levels and/or increments in cellular activity, plus the presence of Cx43 hemichannels on the cell surface, would affect cell survival (Salas et al., 2015). On the other hand, Reactive oxygen species (ROS) and the resulting oxidative damage are involved in the pathophysiology of different types of cataracts (Berthoud and Beyer, 2009; Beebe et al., 2010). LA can induce cell death by opening hemichannels both, directly or indirectly through increases in ROS production, because it has been suggested that free radicals modulates the activity of Cx43 and C46 hemichannels (Retamal, 2014). Independently of the mechanism, the resulting uncontrolled hemichannels opening, induced by LA, would increase ROS production, lead to Ca^2+^ overload and causing the release of important metabolites such as ATP (Retamal et al., 2015). Recently was reported that free radical scavenger Oxyresveratrol, protected human lens epithelial cells of both H_2_O_2_-induced oxidative stress and apoptosis, through the activation of Akt/oxygenase-1 pathway (Hu et al., 2019). The oxidative stress, induce by H_2_O_2_, activates Cx50 hemichannels in fiber cells derived from the chick embryo lens, an effect that is reduced by CBX (Shi et al., 2018). However, our results show that a similar concentration of H_2_O_2_ reduces viability of HLE-B3 cells, which was not prevented by CBX. In fiber cells from chick lens, hemichannels activity protected cells against apoptosis, since mutants that impaired function of Cx50-hemichannels, but not GJC, leads to cell death (Shi et al., 2018). Together, our results are in line with other evidences, suggesting a possible contribution of Cx43 hemichannels to the HLE-B3 cell death induced by LA.

HLE-B3 cells have been widely used as a model for *in vitro* studies of lens epithelial cell physiology, eye-related toxicology and cataracts (Andley et al., 1994; Hosler et al., 2003; Kalariya et al., 2010; Mok et al., 2014). Although it is well-known that transformation markedly alters protein expression pattern in immortalized HLE-B3 cells (Wang-Su et al., 2003), our data support the idea that these cells are a good model to study the role of GJCs and Cx hemichannels in the physiology and pathophysiology of the lens.

## Conclusion

We found that HLE-B3 cells are sensitive to extracellular LA, which diminishes its viability. This effect is related to the ability of LA to open the functional hemichannels mainly formed by Cx43 in the plasma membrane of these cells. The activity and regulation of hemichannels formed by Cx might be an important molecular target to consider in order to study the physiology and pathophysiology of lens cells. Finally, we believe that HLE-B3 cells represent an excellent tool to develop pharmacological test to study biologically significant lens disfunctions.

## Author Contributions

VF and MR contributed to the study conception and the design. VF, OJ, CO, ME, FE, MR, and AV contributed to the data acquisition. VF, AV, GA, and AM contributed to the analysis and data interpretation. VF, AV, CO, MR, and GA drafted the manuscript. AM, GA, and MR contributed to the critical revision of the manuscript.

## Conflict of Interest

The authors declare that the research was conducted in the absence of any commercial or financial relationships that could be construed as a potential conflict of interest.

## Abbreviations

Cx: connexin
GJC: gap junction channel
HLE: human lens epithelial
