# Corrigendum: Contribution of Connexin Hemichannels to the Decreases in Cell Viability Induced by Linoleic Acid in the Human Lens Epithelial Cells (HLE-B3)

**DOI:** 10.3389/fphys.2020.00072

**Published:** 2020-02-11

**Authors:** Vania A. Figueroa, Oscar Jara, Carolina A. Oliva, Marcelo Ezquer, Fernando Ezquer, Mauricio A. Retamal, Agustín D. Martínez, Guillermo A. Altenberg, Aníbal A. Vargas

**Affiliations:** ^1^Instituto de Ciencias Biomédicas, Facultad de Ciencias de la Salud, Universidad Autónoma de Chile, Santiago, Chile; ^2^Instituto de Ciencias de la Salud, Universidad de O'Higgins, Rancagua, Chile; ^3^Department of Pediatrics, University of Chicago, Chicago, IL, United States; ^4^Centro de Envejecimiento y Regeneración (CARE-UC), Departamento Biología Celular y Molecular, Facultad de Ciencias Biológicas, Pontificia Universidad Católica de Chile, Santiago, Chile; ^5^Centro de Medicina Regenerativa, Facultad de Medicina, Clínica Alemana Universidad del Desarrollo, Santiago, Chile; ^6^Universidad del Desarrollo, Centro de Fisiología Celular e Integrativa, Facultad de Medicina Clínica Alemana, Santiago, Chile; ^7^Department of Cell Physiology and Molecular Biophysics, Texas Tech University Health Sciences Center, Lubbock, TX, United States; ^8^Center for Membrane Protein Research, Texas Tech University Health Sciences Center, Lubbock, TX, United States; ^9^Centro Interdisciplinario de Neurociencia de Valparaíso, Instituto de Neurociencia, Facultad de Ciencias, Universidad de Valparaíso, Valparaíso, Chile

**Keywords:** lens, connexin, polyunsaturated fatty acids, cell death, hemichannels

In the published article, there was an error regarding the affiliation for “Aníbal A. Vargas.” As well as having affiliation “9,” he should also have “Instituto de Ciencias de la Salud, Universidad de O'Higgins, Rancagua, Chile.”

Further, “Aníbal A. Vargas” should not have affiliation 1. The affiliation list has been corrected.

The authors apologize for this error and state that this does not change the scientific conclusions of the article in any way. The original article has been updated.

