# Supplementary figures and images for: Contribution of Connexin Hemichannels to the Decreases in Cell Viability Induced by Linoleic Acid in the Human Lens Epithelial Cells (HLE-B3)
Source: Front Physiol. 2020 Jan 20;10:1574. doi: 10.3389/fphys.2019.01574 (PMC6984129; doi:10.3389/fphys.2019.01574)

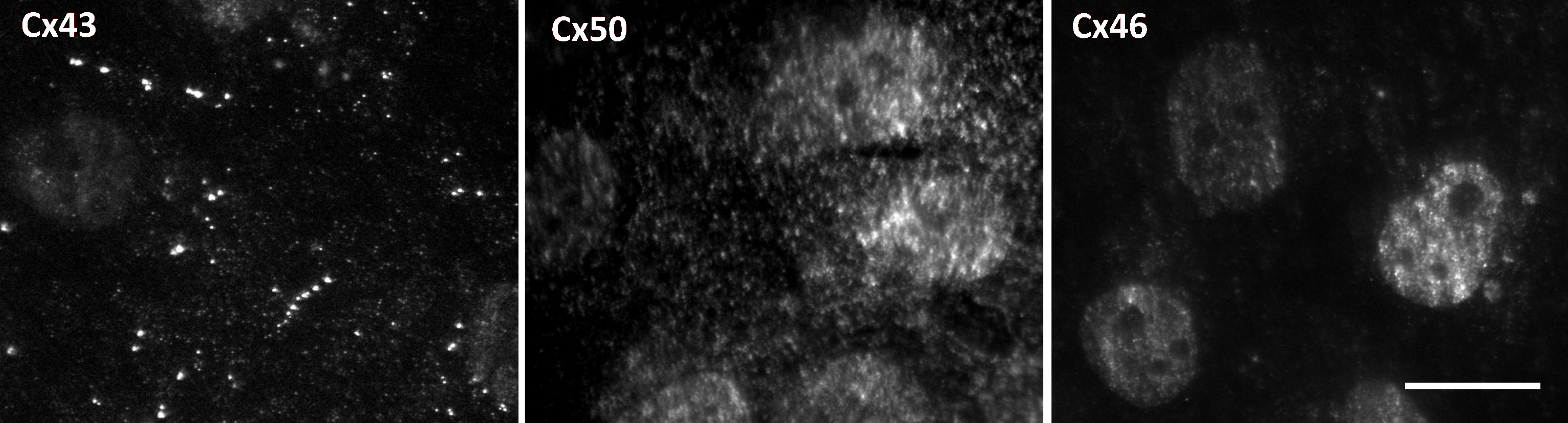

Supplement: Supplementary file 1 [file Image_1.JPEG]

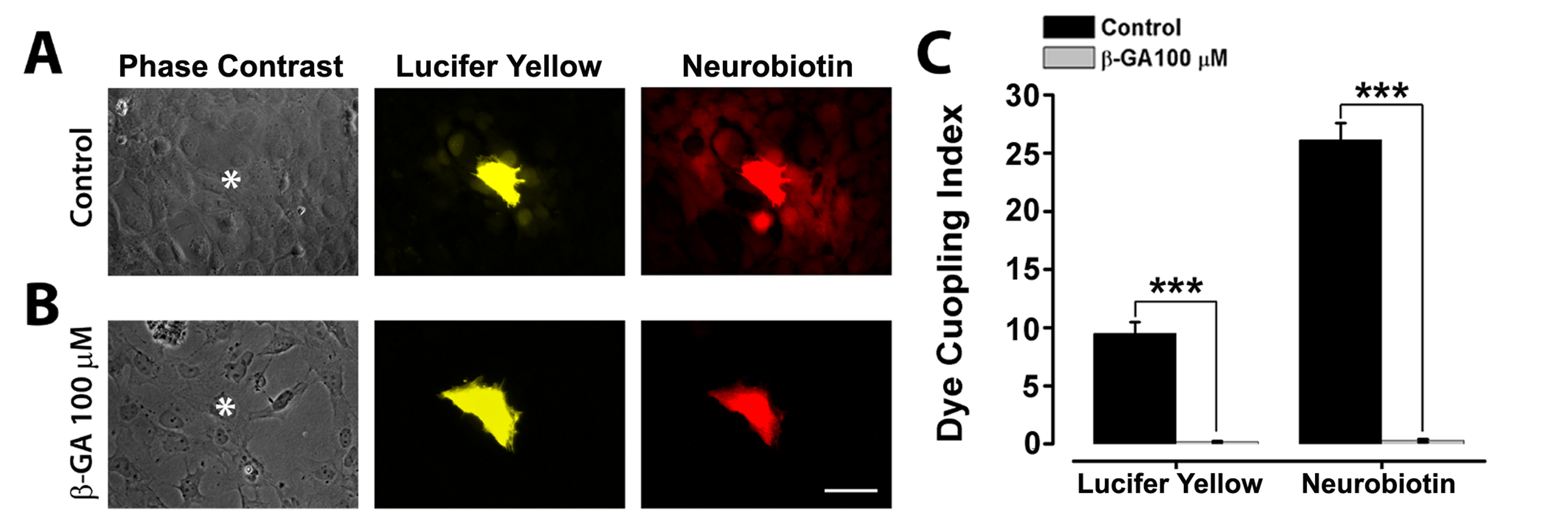

Supplement: Supplementary file 2 [file Image_2.TIF]
