# Supplementary material for: Contribution of Connexin Hemichannels to the Decreases in Cell Viability Induced by Linoleic Acid in the Human Lens Epithelial Cells (HLE-B3)
Source: Front Physiol. 2020 Jan 20;10:1574. doi: 10.3389/fphys.2019.01574 (PMC6984129; doi:10.3389/fphys.2019.01574)
Supplement: Supplementary file 3 [file Table_1.DOCX]

**Supplementary methods**

1. **TIRF microscopy images.** Cx43 fluorescence was identified on HLE-B3 cells using an inverted microscope (Eclipse Ti-E, Nikon, Tokyo, Japan) with a 100 Plan APO TIRF objective (Nikon, Tokyo, Japan) and a Perfect Focus Unit TI-ND6-PFS (Nikon, Tokyo, Japan). Images were acquired with a Digital Camera [C11440](https://www.ncbi.nlm.nih.gov/nuccore/C11440) (ORCA-FLASH 2.0; Hamamatsu Photonics, Hamamatsu City, Japan) and the NIS-Element viewer 4.3 software (Nikon, Tokyo, Japan).
2. **Dye coupling assay.** The functional state of GJCs was evaluated as described by Jara et al. (2012), with minor modifications. Briefly, HLE-B3 cells were grown on coverslips until reaching 80% confluence and then a single cell was microinjected using microcapillaries (Femtotips II, Eppendorf) filled with a solution of 4% Lucifer yellow (LY, MW 457, net charge -2, Sigma-Aldrich) and 4% Neurobiotin (NB, MW 343, net charge +1, Vector Lab) in water with 0.2 to 0.3 s pulses of 1–2 psi for 1–2 min using an InjectMan-FemtoJet system (Eppendorf, Hamburg, Germany). 18-β glycerrithenic acid, a gap junction blocker, was applied in the extracellular media at a final concentration of 100 μM. All microinjections were performed in F-12 medium HCO 3 -free buffered with 10 mM HEPES, pH 7.4 and containing 200 μM lanthanum (La 3+ ) in the extracellular solution to avoid any possible cellular leakage of LY or NB via HCs. Fluorescent cells were observed under ultraviolet illumination using an inverted microscope equipped for epifluorescence and digital microscopy (Nikon TE-2000U, Tokyo, Japan). For NB visualization, cells were fixed with 4% paraformaldehyde in PBS for 20 min, permeabilized with methanol/acetone (1:1) for 2 min at room temperature, and incubated with streptavidin-Cy3 conjugate (Jackson InmunoResearch). Microscopy images were obtained using a Nikon DS-2WBc fast-cooled monochromatic digital camera coupled to the microscope. Dye coupling index was calculated as the mean number of cells to which dye spread occurred.

**Supl. Fig 1:** **Immunolocalization of Cxs in HLE-B3 cells**. Cx43, Cx46 y Cx50 were identified in HLE-B3 cells by TIRF microscopy. The punctate staining denote Cx43 GJC plaques between neighbourhood cells, while Cx46 and Cx50 do not show a similar pattern on the membrane cell. Scale bar 25 µm

**Supl. Fig 2:** **Functional Gap Junction Channels in HLE-B3 cells.** Gap junction communication, expressed as dye coupling index, was evaluated in confluent cultures of HLE-B3 cells. (A) Photomicrographs showing the fluorescent field of HLE-B3 cells microinjected with LY and NB. The transference to the adjacent cells was evaluated under control conditions (LY and NB). (B) Photomicrographs showing HLE-B3 cells in the presence of 100 μM β-GA (LY and NB + β-GA). (C) Quantitative analysis of dye coupling between HLE-B3 cells in both conditions. Bar graphs summarize the results of 3 independent experiments for each condition. Values correspond to the mean ± SEM of fluorescent cell coupling index. ***P &lt; 0.001. Scale bar 10 µm.
